# Feeding modalities, HIV transmission and its predictors among HIV-exposed infants visited Gamo and Gofa zones public health facilities, Southern Ethiopia: a retrospective follow up study

**DOI:** 10.1186/s12887-024-04894-w

**Published:** 2024-06-26

**Authors:** Nigus Kabtu Belete, Nega Degefa Megersa, Sultan Hussen Hebo, Megbaru Debalkie Animut, Eshetu Zerihun Tariku

**Affiliations:** 1 School of Public Health, Arba Minch University, Arba Minch, Ethiopia; 2 School of Nursing, Arba Minch University, Arba Minch, Ethiopia; 3 Department of Public Health and Primary Care, Ghent University, Ghent, Belgium

**Keywords:** HIV-exposed infants, Feeding modality, HIV-transmission, Risk factors, Ethiopia

## Abstract

**Background:**

Despite the highest (88%) Prevention of Mother-To-Child Transmission (PMTCT) of HIV coverage in Eastern Africa, 50% of new HIV infections in children aged 0–14 years occur in the region.

**Objective:**

The aim of this study was to assess the feeding modalities, the rate of HIV transmission and its predictors among HIV exposed infants (HIV-EIs) visited Gamo and Gofa Zones public health facilities, Southern Ethiopia from January 2013 to February 2019.

**Method and materials:**

Institution-based retrospective follow up study was employed among 450 HIV-EIs having DNA/PCR test results. All infant-mother pair records in selected health facilities were reviewed using a standard data extraction tool from March to July 2019. HIV transmission probabilities were assessed by Kaplan–Meier time-to-event analysis method and log-rank tests were used to compare the risk among different groups. The Cox-proportional hazards model, adjusted on infant feeding modalities and other co-variants was used to identify predictors of HIV transmission, and statistical significance was declared at a *p*-value of < 0.05.

**Results:**

In total, 383 complete records were analyzed. In the study, 85.6% (95%CI: 81.6%, 89.1%) of HIV-EIs were exclusively breastfed in the first six months. The 18 months probability of infant HIV transmission was 64 (16.7%) (95%CI: 13.1%–20.8%). The risk of HIV-transmission was higher among infants who were delivered at the hospital than health centers/health posts (AHR = 3.07; 95%CI: 1.19, 7.95); discontinued Cotrimoxazole prophylaxis in at least one visit (AHR = 6.32; 95%CI: 3.35, 11.94); did not exclusively breastfeed (AHR = 3.07; 95%CI: 1.72, 5.47) and came from urban areas (AHR = 5.90; 95%CI: 1.40, 24.85).

**Conclusions:**

The study showed that HIV-EIs had a greater rate of 18 months HIV transmission than the national pooled prevalence. The risk of transmission is higher among infants who do not breastfeed exclusively for the first 6 months, and the risk increases with the number of months spent by breastfeeding. Therefore, strengthening counselling on safer feeding options and Cotrimoxazole prophylaxis use; provision of quality PMTCT service with special focus in hospitals and urban residents were recommended.

## Introduction

Appropriate infant feeding is critical to child survival because the natural food for infants is breast milk. Breastfeeding is one of the foundations of child health, development and survival, especially where diarrhea, pneumonia and under-nutrition are common causes of mortality among children younger than five years [1, 2].

Infants born from Human Immune-Deficiency Virus (HIV) infected mothers are considered as HIV exposed infants. Mother-to-child transmission (MTCT) of HIV infection can occur during pregnancy, labor or delivery, or through breastfeeding. In the absence of any intervention, the risk of HIV infection transmission through breast milk approach 5- 20%. Breast milk substitute has the benefit of zero HIV transmission but carries with it the risk of increased morbidity and mortality from malnutrition, diarrhea and pneumonia [3, 4].

The 2016 World Health Organization (WHO) recommendations on HIV and infant feeding states that mothers living with HIV should breastfeed for at least 12 months and may continue breastfeeding for up to 24 months or longer while being fully supported for Anti-Retroviral Therapy (ART) adherence [2].

Globally, in 2016 an estimated 36.7 million people were living with HIV among those, 2.1 million were children under 15 years of age. Sixty seven percent of the world’s child populations living with HIV live in Eastern and Southern Africa. Each day approximately 5,000 people were newly infected with HIV and approximately 2,800 people died from Acquired Immune Deficiency Syndrome (AIDS). In the same year, 79,000 children became infected with HIV in Eastern and Southern Africa accounting for 49% of the world’s new infections in children while 59,000 children died of AIDS-related causes in the region. This also accounts for 49% of the world’s child AIDS-related deaths [5].

Based on the Federal HIV/AIDS Prevention and Control Office (HAPCO) and the Ethiopian Public Health Institute (EPHI) report, by 2018 a total of 50,923 children aged 0–14 years were living with HIV with an estimated number of 1,055 new HIV infections. In the report, In the South Nation Nationalities and Peoples Representatives (SNNPR) region, 5,667 children were living with HIV with new HIV infection of 100 children [6].

The risk of postnatal HIV infection transmission through breastfeeding is associated with clinical, immunological and virologic maternal factors and infant feeding patterns [3, 4, 7]. Transmission can take place at any point during breastfeeding, and the longer the duration of breastfeeding, the greater the cumulative additional risk. The cumulative probability of late postnatal transmission at 18 months is 9.3% (95% confidence interval, 3.8–14.8%). Late postnatal transmission, therefore, could contribute as much as 42% to the overall rate of MTCT and the transmission risk is around 1% per month of breastfeeding and is constant over time from between four and six weeks to 18 months [4]. Different studies from Ethiopia also affirmed the presence of significant percent of (6- 15.7%) postnatal HIV transmission among HIV exposed infants [8–14]. Feeding modalities were also shown to have an effect on child growth pattern in children born to HIV-infected mothers [15].

During 2015 WHO Guideline Meeting on HIV and infant feeding, systematic reviews provide evidence relating to HIV free-survival to breastfeeding modality. There is strong evidence for HIV-infected women to exclusively breastfeed for the first 6 months but, the decision on continued breastfeeding remains unclear and requires further evidence. There is insufficient information available to estimate the exact association between duration of breastfeeding and the timing of transmission. Evaluating the optimal duration of breastfeeding is the main area to be considered in order to help policy makers choose the most feasible, cost effective ways to reduce vertical transmission and promote the development and growth of children of HIV positive mothers [1, 4, 16].

Therefore, this study is designed to assess feeding modalities and predictors of HIV- transmission among HIV- exposed infants visited Gamo and Gofa Zone health facilities, Southern Ethiopia.

## Methods and materials

### Study setting, design, population and period

Retrospective cohort study was conducted at public health facilities of Gamo and Gofa Zones. During the time of data collection, in the zones, there were five hospitals, 73 health centers, and 471 health posts. The total population of the study area is 2,019,687. According to Gamo and Gofa Zones health departments report, apart from other services, 3 hospitals and 17 health centers were providing ART and Prevention of Mother to Child Transmission (PMTCT) service.

A health post is the lowest level which is staffed by two women each to care for their local populations. These posts offer many services to their community in addition to their focus on mother and child health. Health centers is the next level where they can do certain surgeries, supply more medications, and some even have doctors on staff. Five health posts are managed by health centers. The next level up from health centers are hospitals, which are primarily located in the nation’s cities and offer intensive medical services [17].

All infants and young children (birth to 18 months of age) born from HIV infected mothers and enrolled to PMTCT service who were tested for HIV by using Deoxyribonucleic acid (DNA) or Polymerase chain reaction (PCR) with negative result during January 2013 and finalizing 18 months follow-up until February 2019 in selected hospitals and health centers in Gamo and Gofa Zones, were recruited for this study. This study was carried out during March to July 2019.

### Sample size determination and sampling technique

The sample size was calculated by considering Predictors of HIV- free survival among HIV- exposed infants by using Epi info7 software. Duration of Breast Feeding (BF) cessation is significant variable that gave the largest sample size, by considering two-sided confidence level of 95%, power of 80% and *P* = 94.7% (Infants ceased BF > 3 months and survived at 18 months) [18]. Thus, 404 mother- infant pair records were included in this study. Finally by adding 10% assumption for incomplete data the final sample size was 444.

Public health facilities those provided PMTCT services incorporating follow-up for HIV-exposed infants during January 2013 to February 2019 were considered. In the study period there were three hospital and 17 health centre providing care for HIV-EIs. Health Facilities were selected purposively based on significant number of patients flow they had. Accordingly, all three hospitals (Arba Minch General Hospital (AGM), Sawula District Hospital (SDH), Chencha District Hopital) and 6 health centers (HC) providing follow-up service for HIV-exposed infants (Sikella HC, Sawula HC, Chencha HC, Birbir HC, Shelle HC, and Gerese HC were included.

According to the baseline assessment we have made, the average number of records for HIV-exposed infants who were completed their 18 months follow-up during 1st January 2013 to 30th February 2019 were around 80 in each hospital and 35 for health centers making a total of around 450 records. Therefore, all infant- mother pair records, based on eligibility criteria, in those selected health facilities were reviewed since they are closer to the calculated sample size.

### Data collection methods

The data was collected using structured data extraction tool programmed with Open Data Kit (ODK). The tools were adapted from the national standard HIV exposed infant follow up formats and PMTCT registration log book, which comprises, socio-demographic characteristics, information on Anti Retro Viral (ARV) prophylaxis for the mother and infant, place of delivery and infant feeding practice, outcome of the DNA/PCR test and some other important variables. Maternal ART follow-up cards were also traced to obtain information on some maternal related variables.

The data were retrieved from all registered HIV- exposed infants who were followed from birth to 18 months and DNA/ PCR tested infant charts documented between 1st January 2013 to 30th February 2019. Medical record of the mother and the corresponding infants were cross checked for the availability of their data and linked with infant records in order to confirm maternal prophylaxis data during the appointment period. One data manager per facility were involved in extraction and linking maternal and infant records; while one ART trained nurse and one ART trained health officer or midwife in each health facility were recruited to collect the data. The investigators supervised and monitored the overall process of data collection in all sites.

### Data quality control

To ensure the quality of the data, one day training/orientation was given for data collectors and supervisors on the instruments, method of data collection, ethical issues and the purpose of the study. The data was collected electronically using ODK survey tool application that minimized errors through different setups made in the software. Intensive supervision of data collection was done by onsite supervisors and investigators. The collected data was checked for errors and consistency before analysis and further cleaned using Excel spreadsheet.

### Data processing and analysis

The cleaned data were exported to Statistical Package for the Social Sciences (SPSS) version 22, software. Descriptive data were presented using means with standard deviations and medians with Inter Quartile Ranges (IQR). The event of interest in survival analysis, 18 months cumulative probability of HIV- transmission, was assessed by Kaplan–Meier time-to-event analysis method and the log-rank tests were used for comparison of HIV-transmission among different groups.

The Cox proportional hazards model, adjusted on infant feeding modalities and other co-variants considered in this study was used to identify predictors of 18 months HIV-transmission in children. Variables with *P* values < 0.25 in bivariate analysis were included as covariates in the final multivariable models. For all statistical tests, two-sided *P* values of less than 0.05 were considered to be statistically significant. The crude and adjusted hazard ratio along with their 95% CIs was estimated to predict the hazard of predictors on the occurrence of HIV-transmission. Model goodness-of-fit was tested using graphical methods based on Cox-Snell residuals and the hazard follows 45° that full fills the model goodness of fit.

Censored cases include: children were lost to follow-up at any point in the first 18 months of life, died, unknown HIV status, no clinical evidence of HIV within 18 months of follow-up (infant didn’t show any sign and symptom of HIV infection at the end of 18 months of follow-up) and those with clinical evidence of HIV within 18 months of follow-up (the infants had manifested some signs and symptoms suggesting HIV infection but tested negative for PCR/DNA test at the end of 18 months follow-up).

## Results

### Description of the study population

#### Study characteristics

In this study, 450 records of HIV exposed infants visiting the selected nine health facilities from January 2013 to February 2019 were initially screened. Finally, 383 records were analyzed for examining the risk of HIV transmission making a response rate of 94.8%. Majority of the records 106 (27.5%) were from AGM.

#### Infants’ characteristics

Nearly half 189 (49.0) of the infants were born in the hospitals. The median age of enrollment of the infants in to the care was 6 weeks with minimum and maximum values of ‘0’ and 64 weeks respectively. Two hundred one (52.5%) infants were male by their sex. The median weight of the child at the enrollment time was 3300g with 25th percentiles of 3000g and 75th percentiles 4000g. Regarding Infant ARV prophylaxis, 184 (48%) of infants took Nevirapine (NVP) for 6 weeks. With regard to immunization status 203 (64.0%) of infants were fully immunized during their follow-up time. The proportion of infants who were discontinued cotrimoxazole prophylaxis at least once throughout follow-up time were 24 (6.3%) (Table 1).

**Table 1:** HIV exposed infant’s characteristics at public health facilities in Gamo and Gofa Zone, Southern Ethiopia from January 2013 to February 2019

Characteristics	Category	Frequency	Percentage
Source of study participants	AGH	106	27.7
	Sawula HC	75	19.6
	Chencha HC/Hospital	73	19.1
	SDH	40	10.4
	Arba Minch_HC	45	11.8
	Birbir HC	23	6.0
	Kolla Shelle HC	15	3.9
	Lante HC	6	1.6
Infant’s place of birth	Hospitals	189	49.0
	Health centers	131	34.0
	Home	61	15.1
	Health post	2	0.1
Age at enrollment in weeks (*n* = 380)	≤ 6 weeks	299	78.7
	7–11 weeks	39	10.3
	> = 12 weeks	42	11.0
Sex of study participants	Male	201	52.5
	Female	182	47.5
Infant’s birth weight (*n* = 326)	< 2500g	15	4.6
	> = 2500g	311	95.4
Infant ARV prophylaxis	None	24	6.3
	NVP daily for 6 days	131	34.2
	NVP for 6wks	184	48.0
	SdNVP-AZT-3TC for 7 days	19	5.0
	SdNVP	11	2.9
	Others^a^	14	3.7
Infant immunization status (*n* = 317)	Fully immunized (took BCG, OPV_1 OPV_2 OPV_3 + measles)	203	64.0
	Missed at least one dose/not fully immunized	114	36.0
Cotrimoxazole prophylaxis use among exposed infants throughout the follow-up time	Infant on cotrimoxazole	359	93.7
	Discontinued at least once	24	6.3

*AGH* Arba Minch General hospital, *HC* Health center, *SDC* Sawula district Hospital, *NVP* Nevirapine, *sdNVP* single dose Nevirapine, *AZT* Zidovudine, *3TC* Lamivudine, *BCG* Bacille Calmette-Guerin, *OPV* Oral Polio Vaccine

^a^NVP for 3 or 12 weeks, AZT for 4 weeks

#### Parent/maternal characteristics

Nearly all infants 378 (98.7%) came to the follow-up with their mothers. Two hundred eighty-six (74.7%) mothers were already on ART before pregnancy; among which only 10 (2.6%) were enrolled out of the facility. However, only 141(36.8%) of fathers to the infants in this study were enrolled in HIV/ART care. Regarding the mothers PMTCT intervention, majority of the mothers 143 (37.3%) were on Tenofovir (TDF) + Lamivudine(3TC) + Efavirenz (EFV) drug regimen while 25 (6.5%) of the mothers received none of the interventions. Based on the data obtained from maternal ART card review; at the time of mothers ART follow-up period, the median age of the mother was 29 years with the minimum and maximum age of 16 years and 60 years respectively. The median (IQR) maternal CD4 count and hemoglobin level were 541 (413 to 729) cells/mm^3^ and 12 (11 to 13) g/dl respectively. Regarding the mothers’ breast condition, 27 (7.8%) of the mothers had one or more type of breast abnormalities in one of the follow-up times (Table 2).

**Table 2:** Parents/maternal baseline characteristics at public health facilities in Gamo and Gofa Zone, Southern Ethiopia January 2013 to February 2019

Characteristics		Frequency	Percentages
Care givers	Mothers	378	98.7
	Not mothers	5	1.3
Care givers residence	Rural	92	24.0
	Semi-urban	21	5.5
	Urban	270	70.5
Parent/mother status	Alive	379	99.0
	died	4	1.0
Mother enrolled in HIV/ART care	Yes	286	74.7
	No	97	25.3
Place of mother enrollment in ART (*n* = 286)	Within the facility	276	96.5
	Out of the facility	10	3.5
Mothers PMTCT intervention	(TDF + 3TC + EVF)	143	37.3
	(TDF + 3TC + NVP)	24	6.3
	AZT + sdNVP + 3TC	44	11.5
	HAART	122	31.9
	None	25	6.5
	Sd NVP	12	3.1
	Others^a^	13	3.4
Mother’s age in years (*n* = 257)	< 24	34	12.4
	25–29	108	39.3
	30–34	83	30.2
	> = 35	50	18.2
Parity (number of children born) in number (*n* = 266)	< = 1	64	24.1
	2–3	137	51.5
	> = 4	65	24.4
Mothers’ marital status (*n* = 271)	Married	261	96.3
	Unmarried/single^b^	10	3.7
Maternal CD4 count (*n* = 224)	> 500 cells/mm3	136	60.7
	499 to 350cells/mm3	54	24.1
	< 350 cells/mm3	34	15.2
Mothers WHO staging (*n* = 268)	Stage 1	261	97.4
	Stage 2 and 3	7	2.6
Mothers Hgb status (*n* = 191)	< 12 g/dl	58	30.4
	> = 12 g/dl	133	69.6
Type of delivery (276)	Cesarean section	15	5.4
	Spontaneous vaginal delivery	261	94.6
Mothers ART initiation before pregnancy (*n* = 277)	Yes	266	96.0
	No	11	4.0
Mothers breast condition (*n* = 348)	Normal	321	92.2
	Presence of abnormalities	27	7.8
Father HIV status (*n* = 259)	Negative	86	33.2
	Positive	173	66.8
Father status (*n* = 340)	Alive	330	97.1
	Dead	10	2.9
Father enrolled in HIV/ART care (*n* = 309)	Yes	141	45.6
	No	168	54.4

*ART* Anti-retroviral therapy, *HIV* Human immune Virus, *PMTCT* Prevention of mother to child transmission, *WHO* World health organization, *HAART* Highly Active Antiretroviral Therapy, *AZT* Zidovudine, *TDF* Tenofovir, *3TC* Lamivudine, *EFV* Efavirenz, *sdNVP* single dose Nevirapine, *NVP* Nevirapine

^a^(AZT + 3TC + EVF, second line, pre-ART

^b^2 divorced, 4 separated, 2 never married, 2 widowed

#### Number of HIV exposed infant’s follow-up times in to the care

**Fig. 1:**
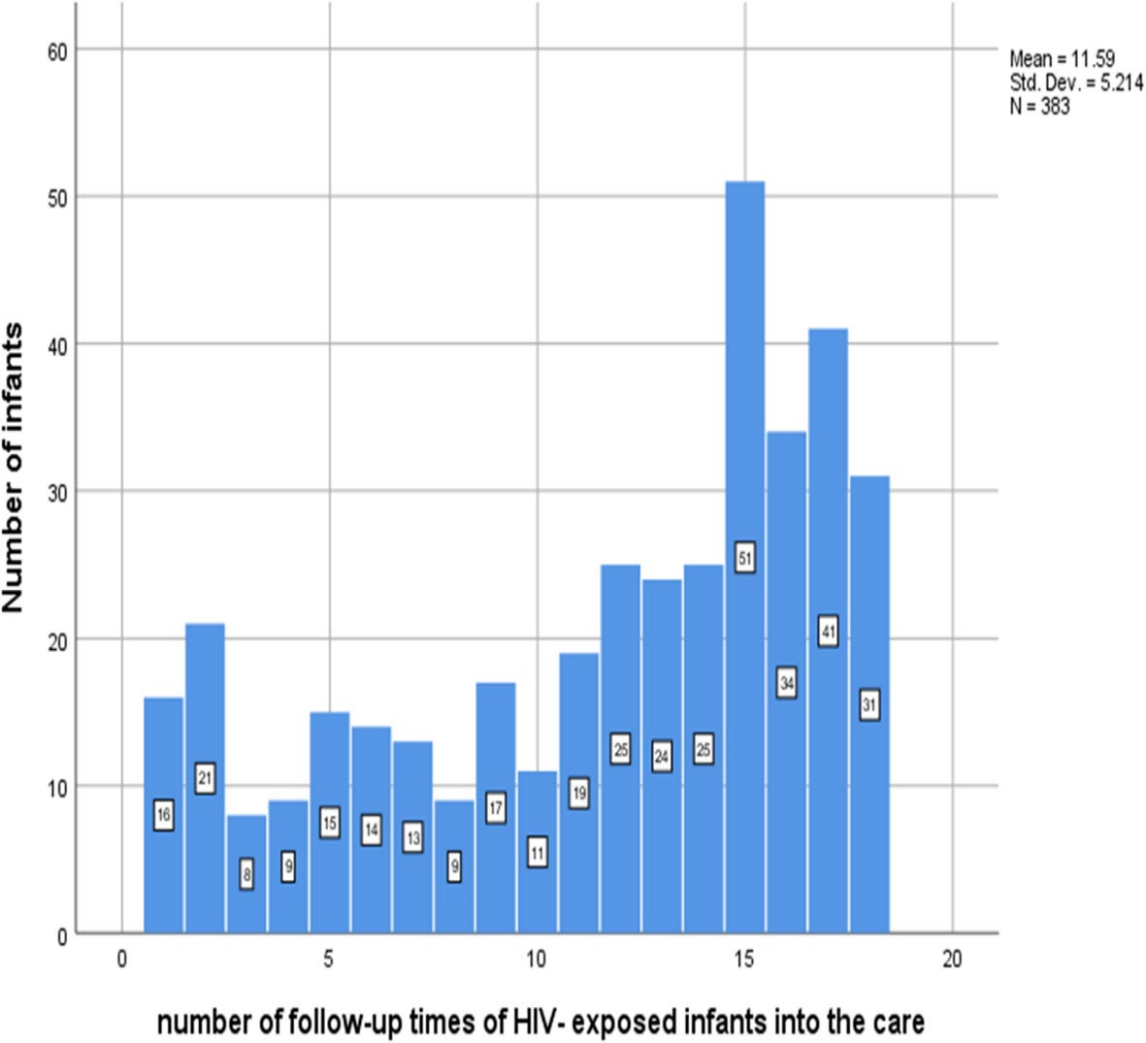
Number of follow-up times of HIV- exposed infants in Gamo and Gofa Zone public health facilities, Southern Ethiopia, January 2013 to February 2019.  Number of follow-up times of HIV- exposed infants

The greater number of infants 51 (13.3%) have 15 follow-up times with minimal and maximum follow-up times of 1 and 18 respectively (Fig. 1).

#### Feeding modalities in the context of HIV-exposed infants

**Fig. 2:**
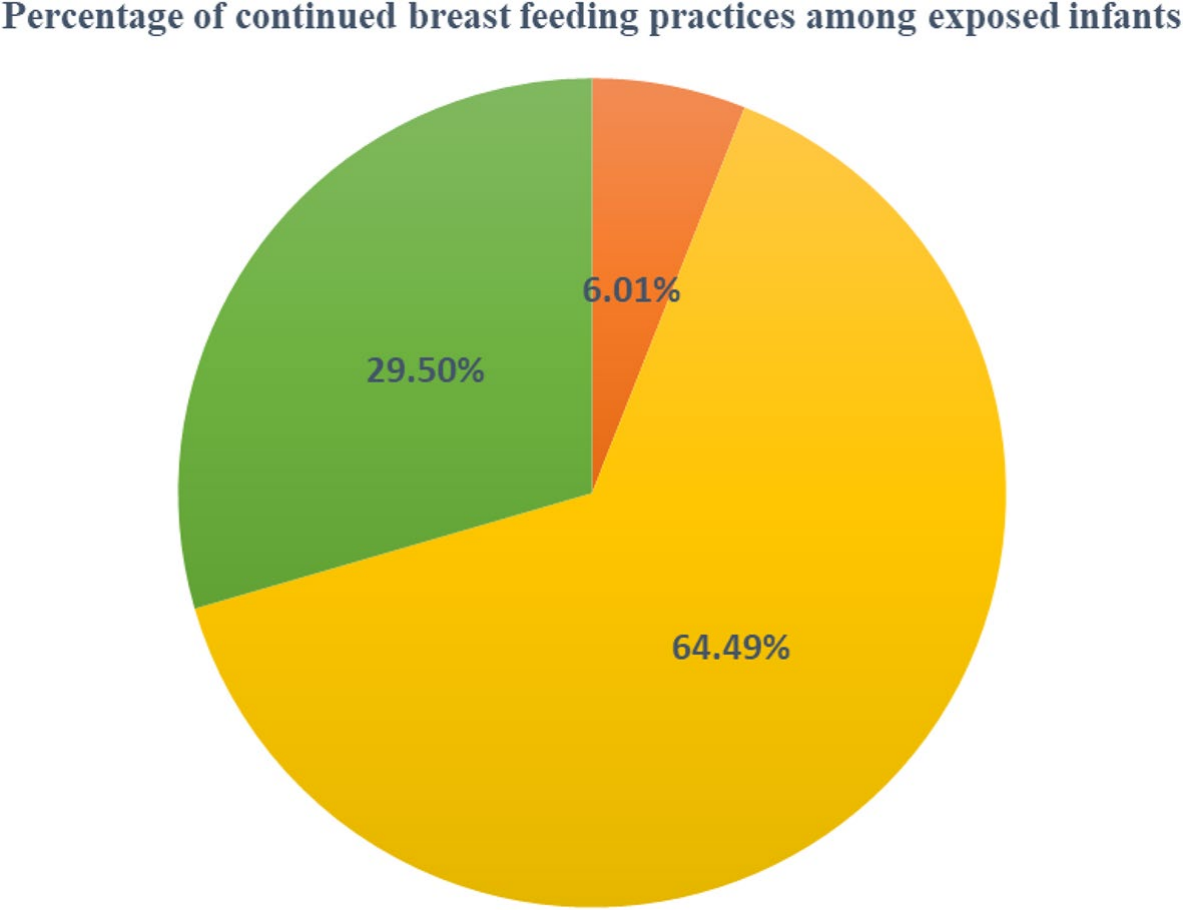
Percentage of feeding modalities of HIV-exposed infants in Gamo and Gofa Zone public health facilities, Southern Ethiopia, January 2013 to February 2019.  Other than non-recommended forms of infant feeding.  EBF for 6 months and continued BF for 13 months or more.  EBF for 6 months and weaned before 13 months

From the total of 362 infants who were enrolled to the care before their 6 months of age, 310 (85.6%; 95%CI: 81.6%-89.1%) of infants Exclusively Breastfed (EBF) for the first six months while 22 (6.1%; 95%CI: 3.8%-9.1%) and 30 (8.3%; 95%CI: 5.7%-11.6%) were on Exclusive Replacement Feeding (ERF) and Mixed Feeding (MF) respectively. Regarding the complementary feeding practice (CFP), 289 (87.3%; 95% CI: 83.2%—90.7%) of infants were on BF with CF; 19 (5.7%; 95%CI: 3.5%-8.8%) were on replacement feeding (RF) with complementary feeding (CF) while 23 (6.9%; 95%CI: 4.5%-10.2%) of infants were weaned off BF immediately after six months of life. When infants were further assessed for the breastfeeding weaning time, among the total of 55 infants weaned off breast feeding with in the 18 months of follow-up time, 24 (43.6%; 95%CI: 30.3–57.7%) of them ceased breast feeding before 13 months of ages while 31 (56.4%; 95%CI: 42.3%-69.7%) of infants weaned off at 52 weeks (13 months) and beyond. The mean (SD) of infants’ breast-feeding weaning time is 49.4 ± 16 weeks (12 ± 4 months). In this study, the number of infants who were on EBF for the first six months and weaned off breast feeding earlier than 13 months of age were only 23 (6.0%; 95%CI: 3.8%-8.9%) (Fig. 2).

#### The cumulative probability of HIV transmission among HIV-exposed infants

In this study about 319(83.3%) infant were censored. Among these 259 (67.6%) were with no clinical or lab evidence, 8 (2.1%) were with clinical evidence, 45(11.7%) were loss to follow up and 7(1.8%) died. This finding also showed that the 18 months (72 weeks) cumulative probability of infant HIV transmission (DNA/PCR test) was 64 (16.7%, 95%CI: 13.1% – 20.8%). The overall proportion of HIV-exposed infants’ mortality was 7 (1.8%); the 18 months cumulative probability of HIV free survival was 259 (67.6%).

**Fig. 3:**
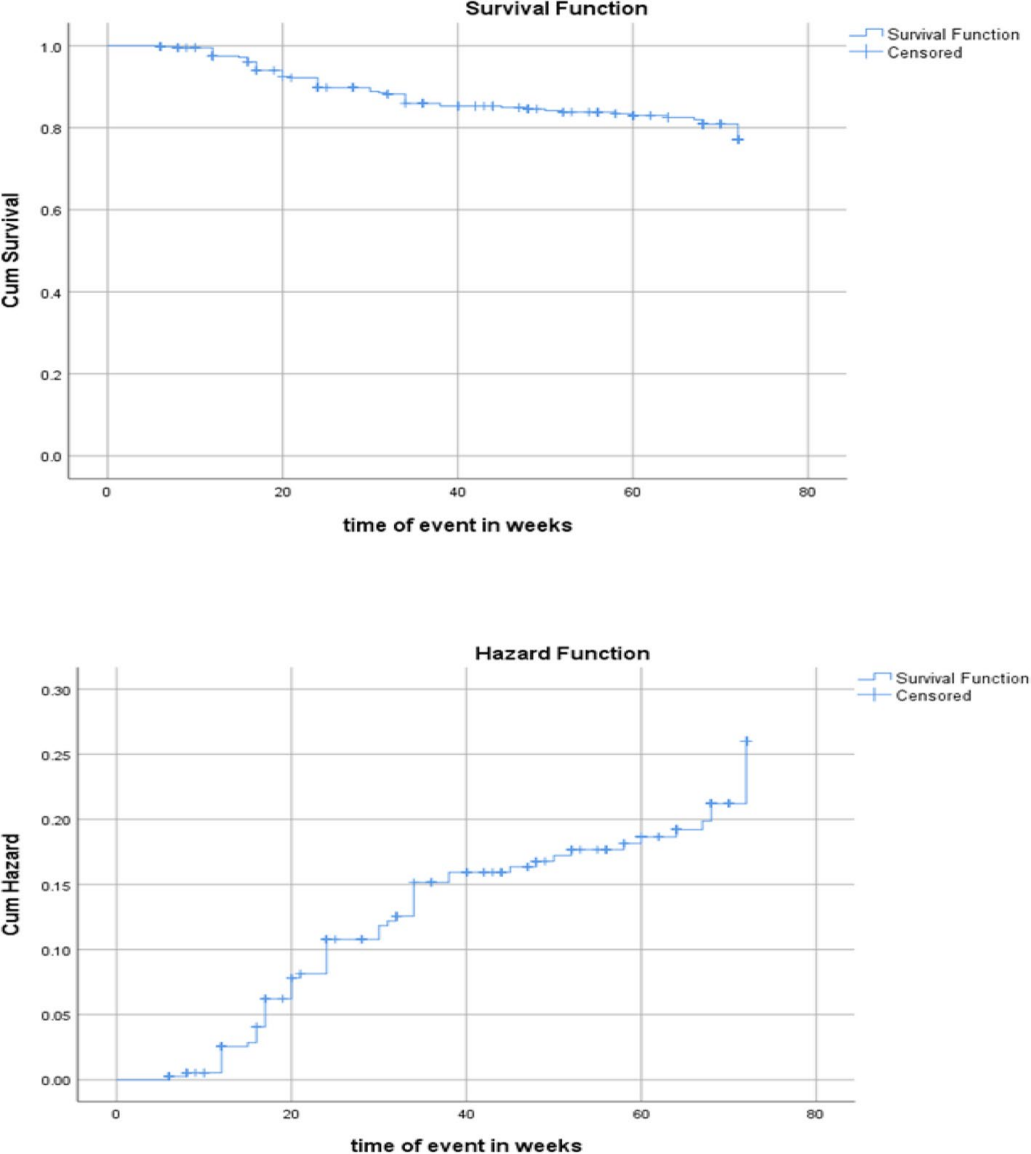
Kaplan–Meier survival and hazard function showing the cumulative probability of infant survival with age.  Survival and hazard function of HIV exposed infants

The total follow-up time was 18,998 weeks (total time at risk) and the median (IQR) time of HIV transmission was 60 (28 to 72) weeks. The study showed an increased probability of acquiring HIV infection as the age of exposed infants increased (Fig. 3).

The study showed no statistically significant difference in cumulative hazard curves for the infant growth status at the end of the follow-up time (Log Rank test = 0.801).

In this study, there is statistically significant difference in cumulative hazard curves for the different infant feeding modalities in the first 6 months of age (Log Rank test = 0.002).

The 18 months probability of infants’ survival curves based on WHO 2016 recommendation for HIV exposed infants (Log Rank test = 0.018).

Infants who were on cotrimoxazole prophylaxis at all visits during the follow-up time have better survival curves compared with those discontinued in at least one visit during the follow-up time (Log Rank test = 0.000).

#### Predictors of HIV transmission among HIV exposed infants

In multivariable cox regression model, HIV exposed infant who were born in the hospitals had three times (AHR = 3.07; 95%CI: 1.19, 7.95) more risk of HIV transmission compared with those delivered at the health center or health post. HIV exposed infants who had discontinued cotrimoxazole prophylaxis in at least one visit had six times (AHR = 6.32; 95%CI: 3.35,11.94) higher risk of HIV transmission when compared with their counterparts those who took cotrimoxazole prophylaxis as per the prescription. Infants who were not on EBF (MF/ERF) were three times more likely to be HIV infected than those who were on EBF (AHR = 3.07; 95%CI: 1.72, 5.47). Infants visiting the health facilities from urban areas were nearly six times (AHR = 5.90; 95%CI: 1.40, 24.85).at greater risk of getting HIV infection compared to those visited the health facilities from rural areas (Table 3).

**Table 3:** Bivariable and multivariable cox repression analysis of infants’ and maternal characteristics that predicted HIV transmission among HIV-exposed infants at Gamo and Gofa Zones public health facilities, Southern Ethiopia

Characteristics		HIV-infection N (%)	Censored N (%)	CHR (95% CI)	*P*-value	AHR (95% CI)
**Variables**	**Category**					
Infant birth place	Health center	12 (9.0)	121 (91.0)	1		1
	Hospital	50 (26.5)	139 (73.5)	2.99 (1.59, 5.61)	0.001	3.07 (1.19, 7.95)^*^
	Home	2 (3.3)	59 (96.7)	0.32 (0.07,1.44)	0.139	0.23 (0.02, 2.31)
Age of enrollment	≤ 6 weeks	50 (16.7)	249 (83.3)	1		
	7–11 weeks	8 (20.5)	31 (79.5)	0.99 (0.47, 2.10)	0.989	
	> = 12 weeks	6 (14.3)	36 (85.7)	0.73 (0.31, 1.70)	0.461	
Sex of infants	Male	35 (17.4)	166 (82.6)	1		
	Female	29 (15.9)	153 (84.1)	0.86 (0.42, 1.4)	0.536	
Infant birth weight (g)	< 2500	3 (20.0)	12 (80.0)	1.01 (0.32, 3.23)	0.986	
	> = 2500g	55 (17.7)	256 (82.3)	1		
Infant ARV prophylaxis	None	3 (12.5)	21 (87.5)	0.70 (0.22, 2.23)	0.544	
	Yes	61 (17.0)	298 (83.0)	1		
Infant immunization status	Missed at least one dose	8 (7.0)	106 (93.0)	0.32 (0.15, 0.68)	0.003	0.46 (0.20, 1.05)
	Fully immunized	48 (23.6)	155 (76.4)	1		1
Cotrimoxazole prophylaxis	Yes (full visit)	47 (13.1)	312 (86.9)	1		1
	DC in at least 1 visit	17 (70.8)	7 (29.2)	6.03 (3.46,10.52)	0.000	6.32 (3.35,11.94)^**^
Infant feeding for < 6 months	EBF	43 (13.9)	267 (86.1)	1		1
	Not on EBF	21 (28.8)	52 (71.2)	2.24 (1.33, 3.77)	0.003	3.07 (1.72, 5.47)^**^
Residence	Rural	5 (4.4)	108 (95.6)	1		1
	Urban	59 (21.9)	211 (78.1)	5.77 (2.32,14.39)	0.000	5.90 (1.40,24.85)^*^
Mother enrolled to ART	Yes	54 (18.9)	232 (81.1)	1		1
	No	10 (10.3)	87 (89.7)	0.55(0.28, 1.08)	0.083	1.06 (0.47,2.40)
Place of enrollment to ART	Out of facility	1 (10.0)	9 (90.0)	0.45 (0.06, 3.29)	0.43	
	Within facility	53 (19.2)	223 (80.8)	1		
Mothers PMTCT intervention	None	7 (28.0)	18 (72.0)	1.85 (0.84, 4.06)	0.125	2.29 (0.43,12.13)
	Yes	57 (15.9)	301 (84.1)	1		1
Mothers breast condition	Normal	56 (17.4)	265 (82.6)	1		
	Abnormality detected	5 (18.5)	22 (81.5)	0.97 (0.39, 2.42)	0.942	

*ARV* Antiretroviral, *DC* Discontinued, *EBF* Exclusive breast feed, *ART* Anti-retroviral therapy, *PMTCT* Prevention of mother to child transmission

^*^p-value < 0.05

^**^p-value < 0.001

## Discussion

The study aimed to assess Feeding modalities, HIV transmission and its predictors among HIV-exposed infants. This study showed that the 18 months cumulative probability of infant HIV transmission was 16.7% (95%CI: 13.1%–20.8%). The result is concordant with the finding reported from Dire Dawa Ethiopia (15.7%) [11]. However, the result is higher than the finding from India (3.7) [13] Botswana (3%) [19]; three African countries (Burkina Faso, Kenya and South Africa) (3.7%) [18]; this might be caused by the widespread use of ART for expectant mothers, elective cesarean sections, and breastfeeding restrictions in other country [20]. But because of limited resources and social standards, these preventative measures are less common in developing nations [21].

Similarly the finding this study is also higher than other studies from sub-Saharan Africa, like Malawi (3.2%) [22]; Zambia (12.8%) [23] and Cameroon (3.59%) [24]. the potential discrepancy might be because of a high level of healthcare awareness and difference in health seeking behaviors mothers.

It is also higher than the pocket studies reported from Ethiopia: 3.8 in Dessie [12]; 10.1% in Gondar [13] [9]; Adama (6.1%) [10]; in Oromia (7.7%) [14] and national pooled prevalence (9.9%) [11]. The higher transmission rate observed in this study might be explained by the difference in the follow-up time (outcome ascertainment time). For instance, the study from Adama Ethiopia revealed HIV transmission rate at 3 months of follow-up which might underestimate the HIV transmission rate. However, as shown in the present study, there was an increased probability of acquiring HIV infection as the age of exposed infants increased. Previous evidences also showed that HIV transmission risk among HIV exposed infants is around 1% per month of breastfeeding and is constant over time from between four and six weeks to 18 months [4, 8]. The data extraction period might also have an impact as the use of 6 years retrospective data in this study might increase the cumulative risk of HIV infection among exposed infants. In addition, the higher transmission rate observed in this study might also warn the slow progress to reduce HIV incidence in Ethiopia. Ethiopia reduced its HIV incidence only by 13.3% between 2010 and 2017 [25]; and reduced MTCT transmission from 39.55% to 16.90% in 2019 as compared to 2000, this level of MTCT is still far too high [26].

Regarding to the feeding modality this study affirmed that 85.6% (95%CI: 81.6%-89.1%) of exposed infants were on EBF for the first six months. The result was much higher than the study findings reported from Nigeria (18.5%) [19]; Kenya (80.4%) [27], and Uganda (29.0%) [28]. It is also higher than reports from different parts of Ethiopia; 30.6% in Addis Ababa [29]; 77.3% in Debre Markos [30], 63.4% in the national and 50.9% sub national pooled prevalence [31]. This variation could be explained by the difference in the study period as breast feeding promotion activities and adherence to the recommended national guidelines are improving from time to time. Moreover, the result in this study is slightly lower than the study from Mekelle were 90.3% of infants were practice EBF [32].

In this study, the magnitude of ERF was 6.1% (95%CI: 3.8%-9.1%). The result was concurred with the study from Debre Markos (8.5%) [30]. However, it is higher than the report from Mekelle Ethiopia were 3.4% of exposed infants were on ERF [32]. On the other hand, the finding is much lower than the result from Nigeria (73.5%) [27], and Addis Ababa, Ethiopia (46.8%) [29]. the lower percentage of ERF practice in the study might be related to the difference in the socio-economic status of the study participants. As replacement feeding inquire the purchase of infant formulas, it is not affordable for most of population groups in Ethiopia who are living in the low socio-economic status which may in turn force the mother/caregiver to rely on breast feeding. The study also revealed that 8.3% (95%CI: 5.7%-11.6%) of infants were on MF which was concurred with the study from Nigeria (8%) [27] and Mekelle, Ethiopia (6.3%) [32]. On the other hand, the result was higher than the study from Kenya (5%) [30]. But lower than the study from Addis Ababa, 15.3% [29], Debre Markos (14.2%) [30], and the national pooled prevalence (23.1%) [31]. MF is identified as one of the major risk factors for HIV transmission among exposed infants; thus, efforts should be taken to further reduce this practice.

Regarding the complementary feeding practices, in the current study, 87.3%; 95% CI: 83.2%—90.7%) of infants were on BF with CF; 5.7% (95% CI: 3.5%-8.8%) were on RF with CF while 6.9% (95%CI: 4.5%-10.2%) of infants were weaned off BF immediately after six months of life. The finding is higher than the studies reported from Adama, Ethiopia were (72%) [10] of infants started their complementary feeding at the age after 6 months. The mean (SD) of infants’ breast-feeding weaning time is 49.4 ± 16 weeks (12 ± 4 months). This finding is in agreement with the study conducted in the southern Ethiopia were the mean duration of breastfeeding among HIV positive mothers was 13.79 months [33]. However, the present study affirmed a prolonged time of breast feeding when compared with the findings from different African countries (Botswana, Burkina Faso, Kenya and South Africa) were greater proportion of infants cease breast feeding in their earlier months of life; with 3–7 months [18, 19]. A relatively longer duration of breast-feeding practice observed in this study might be related to: The positive cultural practices towards breast feeding in Ethiopia; Fear of stigma and discrimination by the mothers as a result of not breast feeding. The percentage with discriminatory attitudes towards people living with HIV among Ethiopian women was 62.7%, SNNPR = 72.3 highest next to Somali region. The presence of stigma and discrimination related to not breast feeding among HIV positive mothers may lead mother to late cessation of breastfeeding of their child [33]. Another possible justification can be the difference in the socio-economic status. This might affect in terms of accessibility of food hygienic conditions that are determinant factors for BF practices, including for EBF.

In reference to WHO 2016 breast feeding recommendation for HIV exposed infants, this study showed lower proportion (6.0%; 95%CI: 3.8%-8.9%) of infants were adhered to the recommendations; EBF for the first six months and weaned off breast feeding at the age of less than 13 months. This variation might be due to the quality of counseling services given on feeding options; although not assessed by this study, there is evidence that HIV positive mothers who have been counseled on feeding options were more likely to adhere to the feeding recommendation as compared to none counseled mothers [31]. Another, possible justification can be the level of knowledge about transmission of HIV from mother to child. In Ethiopia only 57% of mothers know about the risk of MTCT by all three means [33].

In the present study, HIV exposed infants who were delivered in the hospitals had three times more risk of HIV transmission than those delivered in health centers or health posts. The possible justification might be most of the got delivery in the hospital as compared to health center and health post and the majority of mothers/study participants who had underlying medical condition were visited the hospital than health centers/health posts (58 out of 147 (39.5%) mothers with CD4 count < 500 cells/m^3^ were visited the hospitals while only 11/34 (26.5%) of mothers with CD4 count < 500 cells/m^3^ were visited the health centers/heath posts). The presence of such underlying medical conditions among HIV infected mothers increased the risk of HIV transmission among infants [22].

This finding is also an indication to further study the health care facilities, mainly hospitals, quality of PMTCT service provision that might predict HIV transmission among exposed infants. This study, unlike other previous studies [11, 12], did not showed home delivery as a risk factor for HIV-transmission among exposed infants.

In settings with a high prevalence of HIV, high infant mortality due to infectious diseases and limited health-care infrastructure, WHO recommends cotrimoxazole prophylaxis for all HIV-exposed infants and children, starting at four to six weeks after birth and maintained until at least six weeks after cessation of risk of HIV transmission and definitive exclusion of HIV infection in infants [34]. In this study, infants those discontinued cotrimoxazole prophylaxis at least once during their follow-up times had more than six times increased risk of HIV transmission during breast feeding than those who had taking cotrimoxazole preventive therapy. This is in agreement with previous study from Oromia, Ethiopia [9]. However, in another Randomized control trial cotrimoxazole prophylaxis provides no benefit for HIV-uninfected infants in countries that are unaffected by malaria [35].

As expected, this study affirmed that infants born to HIV positive mothers who were not exclusively breast fed (who had MF/ERF) during their first 6 months were three times at greater risk of acquiring HIV than their counterparts who had only breastfed in their first 6 months. This is consistent with the study from Cameroon [24] and studies from different parts of Ethiopia [10, 11, 13, 14]. This could be because mixed feeding is associated with gastrointestinal ulceration secondary to diarrheal disease. As a result, the virus can quickly enter the infant’s bloodstream through the ulcerated gastrointestinal tissue. However, the study did not show significant difference in HIV transmission by infant feeding modality in later years of child (neither feeding as per WHO recommendation nor infant BF weaning time). This might be related to the minimal number of infants that were adhered to the WHO feeding recommendation in this study. The unadjusted analysis showed that infants those exclusively breastfed for the first 6 months and weaned of BF before 13 months had a better cumulative survival (Log Rank test = 0.018) which also supported by the study from Malawi where risk of late postnatal transmission (occurring after 6 months) was higher in women who had evidence of breastfeeding beyond 6 months [22].

Place of residency is one of the determinant factors to access health service utilization. In majority of the cases, it is very clear that individuals residing in rural areas have lower access to the health services than those residing in urban areas. However, in the present study, the risk of HIV transmission was nearly six times higher among those infants visited the health facilities from urban area when compared with their counter parts those came from rural area. This can be explained by: in the case of ART, PMTCT, and related services the scenario might be different as those people living with HIV do like to visit the health facilities that are far from their usual resident area due to fear of stigma and discrimination. In this study, for instance, among 97 mothers who were not enrolled in ART service significant numbers 81(83.3%) of them were from urban residents. Similarly, among 25 mothers those who didn’t receive PMTCT intervention, more than 50% of them [11] were residents of urban area.

This study is not supported by other studies from Dire Dawa district which stated that rural residence were three times at higher risk of acquiring HIV infection than those born to mothers from urban areas [36]. It could be due to difference in health care service utilization and due to presence of different non-governmental organization which gave more emphasis on the rural population.

## Strength and limitations of the study

The major strength of this study was the longer follow-up time as fewer studies have reported on 18 months outcomes of HIV exposed infants. The measurement of infant feeding modalities was also done at each follow-up time that will minimize the risk of recall bias.

One of the limitations of the study was both mothers and infants do not have data at all‐time points, thus the missing data limited the inclusion of some relevant predictor variables in the survival analysis. However, efforts were made to manage the missing values. There was some loss to follow‐up, which could result in biased estimates of HIV transmission later in life.

## Conclusions

A higher rate of 18 months HIV-transmission among exposed infants compared to the national pooled prevalence was observed. Similarly there were significant numbers of infants were on EBF in the first 6 months of life. However, less than ten in hundred infants were ceased breast feeding at the age of 13 months or younger. The risk of HIV-transmission is higher among infants delivered at the hospitals, discontinued cotrimoxazole prophylaxis at least once, non-exclusively breastfed in the first 6 months and those who reside in urban areas.

Therefore HIV-infected mothers those reside in urban area would improve their health seeking behaviors to receive proper ART and PMTCT intervention to increase the probability of their infant’s survival.

Program planners and health professionals should strengthen continuous counseling on safer feeding options that would to further reduce the risk of late post-natal transmission, Monitoring and evaluation of policy/guideline implementation regarding infant feeding modalities.

Future research should attempt to replicate this study with prospective follow-up study mainly to drug adherence and have anthropometric data at necessary time points and predict the growth trajectory among the different feeding modalities as it an important parameter to decide on the exposed infants feeding modalities.

## Data Availability

The original data for this study is available from the corresponding author.

## Abbreviations

AGH: Arba Minch General Hospital
AIDS: Acquired Immune Deficiency Syndrome
ART: Anti-Retroviral Therapy
ARV: Anti-Retroviral
BF: Breast feeding
CF: Complementary Feeding
DNA: Deoxyribonucleic acid
EDHS: Ethiopian Demographic and Health Survey
EPHI: Ethiopian Public Health Institute
EBF: Exclusive Breastfeeding
ERF: Exclusive Replacement Feeding
HAPCO: HIV/AIDS Prevention and Control Office
HIV: Human Immune-deficiency Virus
HIV–EIs: Human Immune-deficiency VirusExposed Infants
HR: Unadjusted Hazard Ratio
IQR: Inter Quartile Range
MTCT: Mother-To-Child Transmission of HIV
MF: Mixed Feeding
NVP: Nevirapine
PCR: Polymerase Chain Reaction
PMTCT: Prevention of Mother-To-Child Transmission of HIV
RF: Replacement Feeding
SDH: Sawula District Hospital
SNNPR: South Nation Nationalities and Peoples Representatives
WHO: World Health Organization
